# Room‐Temperature Nanoseconds Spin Relaxation in WTe_2_ and MoTe_2_ Thin Films

**DOI:** 10.1002/advs.201700912

**Published:** 2018-04-14

**Authors:** Qisheng Wang, Jie Li, Jean Besbas, Chuang‐Han Hsu, Kaiming Cai, Li Yang, Shuai Cheng, Yang Wu, Wenfeng Zhang, Kaiyou Wang, Tay‐Rong Chang, Hsin Lin, Haixin Chang, Hyunsoo Yang

**Affiliations:** ^1^ Department of Electrical and Computer Engineering, and NUSNNI National University of Singapore Singapore 117576 Singapore; ^2^ Center for Joining and Electronic Packaging State Key Laboratory of Material Processing and Die & Mould Technology School of Materials Science and Engineering Huazhong University of Science and Technology Wuhan 430074 China; ^3^ Department of Physics National University of Singapore 2 Science Drive 3 Singapore 117542 Singapore; ^4^ Centre for Advanced 2D Materials and Graphene Research Centre National University of Singapore 6 Science Drive 2 Singapore 117546 Singapore; ^5^ Institute of Physics Academia Sinica Taipei 11529 Taiwan; ^6^ SKLSM Institute of Semiconductors Chinese Academy of Sciences P. O. Box 912 Beijing 100083 China; ^7^ Department of Physics National Cheng Kung University Tainan 701 Taiwan

**Keywords:** 2D materials, spin dynamics, spin polarization, time‐resolved Kerr rotation, Weyl semimetals

## Abstract

The Weyl semimetal WTe_2_ and MoTe_2_ show great potential in generating large spin currents since they possess topologically protected spin‐polarized states and can carry a very large current density. In addition, the intrinsic non‐centrosymmetry of WTe_2_ and MoTe_2_ endows with a unique property of crystal symmetry‐controlled spin–orbit torques. An important question to be answered for developing spintronic devices is how spins relax in WTe_2_ and MoTe_2_. Here, a room‐temperature spin relaxation time of 1.2 ns (0.4 ns) in WTe_2_ (MoTe_2_) thin film using the time‐resolved Kerr rotation (TRKR) is reported. Based on ab initio calculation, a mechanism of long‐lived spin polarization resulting from a large spin splitting around the bottom of the conduction band, low electron–hole recombination rate, and suppression of backscattering required by time‐reversal and lattice symmetry operation is identified. In addition, it is found that the spin polarization is firmly pinned along the strong internal out‐of‐plane magnetic field induced by large spin splitting. This work provides an insight into the physical origin of long‐lived spin polarization in Weyl semimetals, which could be useful to manipulate spins for a long time at room temperature.

The emerging 2D transition metal dichalcogenides (TMDs), due to their strong spin splitting in d orbits and valley momentum separation,^1^ provide a fertile ground to explore the spin and valley degrees of freedom.^2^, ^3^ The intrinsic inversion symmetry breaking in monolayer hexagonal 2H‐TMDs (MX_2_ with M = W, Mo and X = Se, S) generates the valley‐contrasting optical selection rules. The spin‐valley coupling reduces spin‐flip scatterings, leading to a long spin and valley relaxation.^4^ However, the semiconducting nature makes it challenging to apply a large current density in 2H‐TMDs, restricting their applications in spintronic devices with typical vertical or lateral device structures of ferromagnet/TMDs.^5^

Recently, the semimetal 2D TMDs, e.g., Weyl semimetal WTe_2_ and MoTe_2_, have sparked intense research interest as they possess topological nontrivial electronic structures in the bulk and Fermi arcs at surface.^6^ In stark contrast to 2H‐TMDs, WTe_2_ and MoTe_2_ crystallize in the orthorhombic structure without centrosymmetry (Td phase).^7^ With large spin–orbit coupling in W(Mo) 5d (4d) orbitals and Te 5p orbitals,^8^ both the bulk and surface Fermi arcs present nondegenerate spin texture.^9^ Interestingly, the intrinsic inversion‐symmetry breaking of WTe_2_ can induce an out‐of‐plane antidamping spin–orbit torque in WTe_2_/ferromagnet heterostructures as the current is applied along the low‐symmetry axis of WTe_2_.^10^ Meanwhile, the semimetallic WTe_2_ and MoTe_2_ have shown a remarkably high current density (≈50 MA cm^−2^),^11^ indicating WTe_2_ and MoTe_2_ are promising candidates for generating large charge‐to‐spin current conversion. Therefore, understanding the underlying physical mechanisms of spin dynamics in WTe_2_ and MoTe_2_ is fundamentally important and technologically useful for semimetallic WTe_2_‐ and MoTe_2_‐based spintronic devices.

Here, we investigate spin dynamics in centimeter‐scale, chemical vapor deposition (CVD)‐grown WTe_2_ and MoTe_2_ thin film by performing the time‐resolved Kerr rotation (TRKR). We find a long‐lived spin polarization up to the nanosecond timescale at room temperature. A physical mechanism based on the first principle calculation of band structure is proposed to explain the long‐lived spin polarization. Moreover, we find that the optically induced spin polarization is robust against an externally applied magnetic field, supported by spin dynamics analytical simulations.

WTe_2_ and MoTe_2_ (space group *P*_mn21_) have an orthorhombic structure consisting of 1D zigzag chains with W(Mo) atoms along the *x* direction (**Figure** **1**a). It possesses a layered structure like other 2D TMDs but with additional distortion of W(Mo) atomic chains along the *y* direction (top panel in Figure 1a), resulting in an inversion symmetry breaking. The spin texture (Figure 1b; Figures S1, S2, and S3a,b, Supporting Information) and the spin–orbit splitting energy (Δ_sp_) (Figure 1c and Figure S3c, Supporting Information) of two lowest conduction bands are estimated from the tight‐binding Hamiltonian interpolated from the ab initio calculation (see Supporting Information Note 4 for details). Due to the requirements of time‐reversal symmetry and lattice symmetry operations (*c*_2_ *_z_*, *σ_zx_*, and *σ_yz_*), the spin polarization along *z* (*S_z_*) and *y* (*S_y_*) in the *k_x_* and −*k_x_* valleys have opposite signs (see Figure 1b; Figures S2a, S3a,b for *S_z_*; Figures S1 and S2b for *S_y_*, Supporting Information). For few‐layers W(Mo)Te_2_, the spin has no *x*‐component for *k* point along *Γ*–*X* because of the mirror symmetry with respect to the *z*–*y* plane (*σ_zy_*). Moreover, few‐layer WTe_2_ and MoTe_2_ share the same inversion asymmetric property as in bulk crystal, which leads to large spin–orbit splitting. The Δ_sp_ along the high symmetry path is defined by *ES*_↑_−*ES*_↓_, where the *ES*_↑_ and *ES*_↓_ indicate the energy bands with up and down spins, respectively. The Δ_sp_ at the bottom of the conduction band of bilayer and 12‐monolayer WTe_2_ and MoTe_2_ reaches 30–45 (Figure 1c) and 15–25 meV (Figure S3c, Supporting Information) respectively, which is comparable to monolayer 2H‐TMDs (Figure S4, Supporting Information). The distinct spin orientation in few layers WTe_2_ and MoTe_2_ indicates the potential spin injection by circularly polarized light excitation.

**Figure 1:**
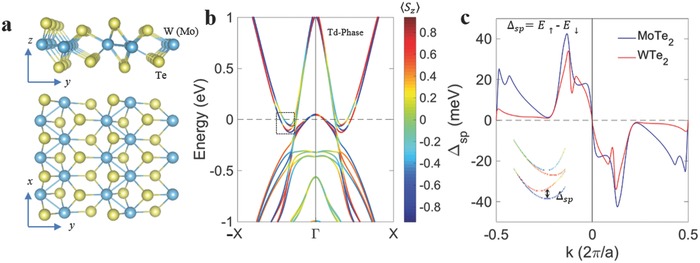
Spin texture and spin–orbit splitting energy of WTe_2_ and MoTe_2_. a) Atomic structure of monolayer WTe_2_ and MoTe_2_. Light blue balls denote W(Mo) atoms. Yellow balls represent Te atoms. W(Mo) atomic chains distort along the *y* direction (top panel). b) Spin texture of bilayer WTe_2_. The color bar in the right label indicates the out‐of‐plane spin polarization ⟨*S_z_*⟩. Due to the requirements of time‐reversal symmetry and lattice symmetry (*σ_zy_*), the spin ⟨*S_z_*⟩ reverses its polarizations at −*k_x_* and *k_x_*. c) The Δ_sp_ as a function of momentum *k_x_* along *Γ–X*. The Δ_sp_ of both WTe_2_ and MoTe_2_ at the bottom of the conduction band reaches ≈40 meV. The inset shows the spin–orbit splitting energy Δ_sp_ on the conduction band because of inversion symmetry breaking. The Δ_sp_ is defined by energy band differences between spin‐up (*ES*_↑_) and spin‐down (*ES*_↓_) electrons. Only the results of bilayer are presented since Δ_sp_ is still obvious in both materials, when the thickness increases to 12 monolayers (Figure S3, Supporting Information).

The investigation of spin dynamics is performed on CVD‐grown WTe_2_ and MoTe_2_ thin films. So far, most of WTe_2_ and MoTe_2_ thin film samples were mechanically exfoliated and thus limited to small flakes.^12^, ^13^ Here, we prepared centimeter‐scale WTe_2_ and MoTe_2_ thin films via CVD (see Experimental Section for details). The WTe_2_ thin film is uniform in large area as shown in optical microscopy (OM) images (**Figure** **2**a). The photograph reveals that the thin film covers the whole substrate of Si/SiO_2_ with the area of 1 × 1 cm^2^ (inset of Figure 2a), which is important for practical applications and optical measurements. The WTe_2_ thin film usually has ≈5–11 monolayers with a typical thickness of ≈6.4 nm measured by atomic force microscopy (AFM) (Figure 2b) for WCl_6_‐derived WTe_2_ (sample 1). The Raman spectra (Figure 2c) show peaks at ≈110, 116, 132, 163, and 211 cm^−1^ corresponding to the A^4^_2_, A^3^_1_, A^4^_1_, A^7^_1_, and A^9^_1_ vibration modes, respectively, which agrees well with that of mechanically exfoliated thin flakes from WTe_2_ single crystal (Figure S6, Supporting Information) and previous reports,^14^ demonstrating the Td‐phase nature of WTe_2_. The Raman mapping data of the A^7^_1_ characteristic peak at ≈163 cm^−1^ confirm that the sample is in the uniform Td crystal structure (inset of Figure 2c). The X‐ray photoelectron spectroscopy (XPS) data in Figure 2d show the chemical states of W 4d and Te 3d electrons. The binding energy of W 4d_3/2_ (256.8 eV), W 4d_5/2_ (244.1 eV), Te 3d_3/2_ (583.7 eV), and Te 3d_5/2_ (573.4 eV) reflects the valence states of W (+4) and Te (−2) in WTe_2_ thin film. X‐ray diffraction (XRD) and high‐resolution transmission electron microscope (HRTEM) results of WTe_2_ are shown in Figure S9a,b in the Supporting Information, respectively. The XRD pattern of WTe_2_ thin film is dominated by diffraction peaks of crystal planes (002*n*, *n* = 1, 2, 3,……),^15^ indicating the thin film orients along the *z*‐axis (Figure S9a, Supporting Information). The HRTEM shows that the WTe_2_ thin film is polycrystalline (Figure S9b, Supporting Information). It consists of multiple single crystallines with a grain size of dozens of nanometers. The lattice distance of (002) planes is ≈0.7 nm.

**Figure 2:**
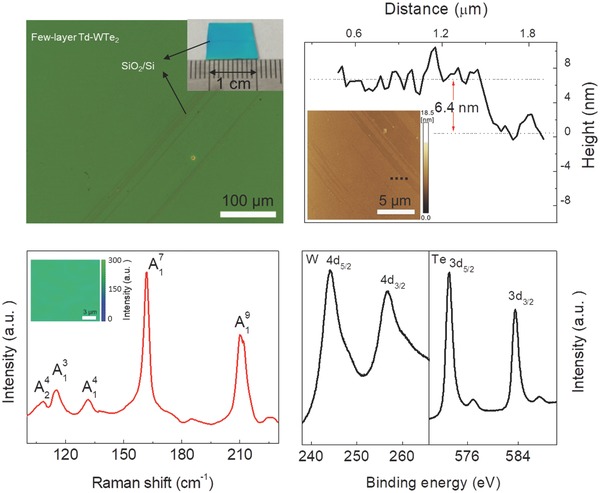
Large‐area CVD‐grown WTe_2_ thin film. a) OM image of a representative few‐layer WTe_2_ thin film. The inset shows a photograph of WTe_2_ thin film on a Si/SiO_2_ substrate. A scratch in the middle of photograph shows the contrast with Si/SiO_2_ substrate. The WTe_2_ thin film covers the whole Si/SiO_2_ substrate with the area of 1 × 1 cm^2^. b) Height profile of WTe_2_ thin film. The inset is the corresponding AFM image. The thickness ranges from 5 to 11 nm (≈5–11 monolayers). c) Raman spectra of a typical WTe_2_ thin film. The peaks located at ≈110, 116, 132, 163, and 211 cm^−1^ corresponds to the A^4^_2_, A^3^_1_, A^4^_1_, A^7^_1_, and A^9^_1_ vibration modes, respectively, which indicates the Td‐phase nature of WTe_2_ thin film. The inset is the Raman mapping integrated from A^7^_1_ peak at 163 cm^−1^, confirming the uniformity of WTe_2_ thin film. d) XPS from W 4d and Te 3d electrons. It shows the binding energy of W 4d_3/2_ and W 4d_5/2_ at ≈256.8 and 244.1 eV, and Te 3d_3/2_ and Te 3d_5/2_ at ≈583.7 and 573.4 eV, respectively. The atomic ratio of Te to W derived from XPS is ≈2 which is consistent with the stoichiometry of WTe_2_.

A similar method (see Note S1, Supporting Information) was applied to prepare few‐layer MoTe_2_ (sample 2) with a thickness of ≈11.4 nm (Figure S7b, Supporting Information) using a molybdenum trioxide (MoO_3_) thin film as a reaction source. The optical microscopy image (Figure S7a, Supporting Information), Raman spectra (Figure S7c, Supporting Information), Raman mapping (Figure S7d, Supporting Information), and XPS (Figure S8, Supporting Information) verify the pure and uniform Td phase of MoTe_2_ thin film.^12^, ^16^ The quantitative analysis derived from the XPS spectra shows that the atomic ratio of Te to W(Mo) in few‐layer WTe_2_ and MoTe_2_ is ≈2, which is consistent with the stoichiometric amount of WTe_2_ and MoTe_2_. The XRD (Figure S9c, Supporting Information) and TEM (Figure S9d, Supporting Information) results confirm that the MoTe_2_ thin film orients along the *z*‐axis. The above two samples were used to perform TRKR.

The experimental TRKR setup is depicted in **Figure** **3**a. Ultrafast laser pulses of a duration of 120 fs and wavelength of 800 nm were generated at a repetition rate of 1 kHz from a Ti: Sapphire oscillator. Each pulse was later split into an intense exciting pump pulse (800 nm) and detecting linearly polarized probe pulse (400 nm). All experiments were performed at room temperature (see Experimental Section). The pump and probe power are 600 and 20 µW, respectively. Figure 3b displays typical TRKR dynamics from sample 1 following the excitation by circularly polarized pump pulses. In the magneto‐optical Kerr effect, the polarization of probe light is sensitive to the magnetization of the magnetic medium. In our work, the normal incident circularly polarized light (800 nm) induces a net out‐of‐plane spin polarization hold by the photogenerated carriers. The net spin polarization acts as a mean magnetization field which rotates the polarization of the probe light (400 nm) after reflection from the surface of WTe_2_ and MoTe_2_ thin films. In order to exclude the interference from the pump light (800 nm) on the Kerr signals, a long‐pass filter was used to filter the pump light before the probe light (400 nm) enter the balanced photodetector (see Experimental Section for the details).

**Figure 3:**
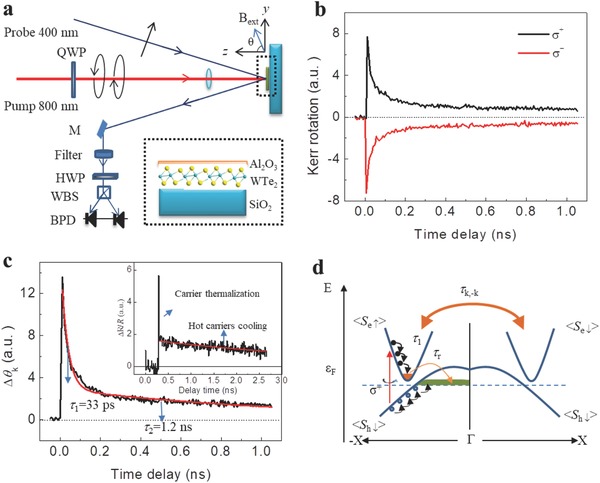
Room‐temperature long‐lived spin polarization. a) Schematic diagram of TRKR setup. We use left‐ (σ^+^) or right‐ (σ^−^) circularly polarized pump pulses to excite spin‐polarized electrons and holes. QWP: quarter wave plate. M: reflectivity mirror. HWP: half wave plate. WBS: Wollaston beam splitter. BPD: balanced photodetector. b) TRKR traces under excitation of σ^+^ and σ^−^ pump. The Kerr rotation changes the sign when the helicity of pump pulse is reversed, indicating the Kerr rotation arises from optically induced spin polarization. c) Signals difference (Δθ_k_) between σ^+^ and σ^−^ pump. Two dominant decay processes (τ_1_ = 33 ps and τ_2_ = 1.2 ns) can be extracted by biexponential fitting. Inset is the ultrafast transient reflectivity. d) Schematic diagram of WTe_2_ band structure with spin relaxation process. The momentum separation between the bottom of the conduction band and the top of the valence band obstructs the recombination of electron–hole pairs. Furthermore, the backscattering between *k_x_* to −*k_x_* is forbidden due to time‐reversal symmetry and lattice symmetry (*σ_zx_* and *c*_2_ *_z_*) operation. The horizontal dashed line shows the position of the Fermi level (ε_F_). ⟨*S*_e↑_⟩ and ⟨*S*_e↓_⟩ denote spin‐up and spin‐down polarization of electrons, respectively, while ⟨*S*_h↑_⟩ and ⟨*S*_h↓_⟩ label spin‐up and spin‐down polarization of holes, respectively.

Figure 3b shows that the Kerr signal changes its sign when the pump beam reverses its helicity (σ^+^ and σ^−^), emphasizing the fact that the light‐induced spin polarization is determined by the pump helicity. As shown in Figure 1b and Figures S1–S3 in the Supporting Information, the semimetallic WTe_2_ and MoTe_2_ show distinct conduction and valence bands with *k* dependent spin splitting compared with that of metals due to the spin–orbit coupling. Here, we consider that the pump causes the transition of electrons from the occupied valence bands (W(Mo) d orbitals) to the empty conduction bands (Te p orbitals) and neglect the intraband transitions. We further consider that the angular momentum is conserved during the transitions. The spin dynamics of WTe_2_ and MoTe_2_ is reflected in the temporal decay of the Kerr rotation.

To avoid spurious experimental artifacts, we apply our analysis to the difference of the signals obtained with σ^+^ and σ^−^ pump pulses, as shown in Figure 3c. The resulting spin dynamics presents two decay processes, which can be estimated by a biexponential fitting. To elucidate the nature of long spin relaxation, we simplify the system to be governed by the spin‐polarized electrons. Similar spin dynamics is observed in sample 2 (Figure S10, Supporting Information). An initial fast exponential decay of τ_1_ = 33–61 ps (*t* < 0.1 ns) is followed by a slower decay of τ_2_ = 0.35–1.2 ns (*t* > 0.2 ns). The variation of lifetimes is likely caused by materials difference (0.35 ns for MoTe_2_ and 1.2 ns for WTe_2_). It is also challenging to keep the electronic properties such as the carrier mobility and density of all thin films due to variable environmental factors in our CVD process. The spin relaxation time thus slightly changes from sample to sample.

Combining the spin texture of WTe_2_ and MoTe_2_ along *Γ*–*X*, we can understand the long spin relaxation as follows. The density functional theory (DFT) simulations (Figure 1b; Figures S2a and S3a, Supporting Information) have shown that the spin polarization is almost out‐of‐plane where the pump light is applied. The time‐reversal and lattice symmetry requires states with the opposite spins at *k_x_* and −*k_x_* valleys. Figure 3d is the schematic of WTe_2_ band structure with spin relaxation process. The Hall transport data (Figure S11 and Note S2, Supporting Information) show that the majority carrier of WTe_2_ thin film is hole, indicating the Fermi level resides in the valance band. Electrons with up‐spins are photogenerated by left‐circularly polarized light (σ^+^) at −*k_x_* due to the conservation of angular momentum during the optical transition. Subsequently, a fast spin depolarization occurs with a characteristic time of 30−60 ps which originates from the carrier–phonon or carrier–carrier scattering in their respective bands at a rate $\tau_{1}^{-1}$. The recombination of electron–hole pairs decreases the spin density of photogenerated carriers causing the decay of Kerr effect signal. However, states at the conduction band and valance band are separated in momentum space (Figures 1b and 3d). Therefore, the momentum conversion must be assisted by phonons, and such a phonon‐assisted process lowers the recombination probability. Our results of ultrafast transient reflectivity suggest a weak phonon scattering in WTe_2_ thin film. As shown in the inset of Figure 3c, subsequently after light excitation, the photo‐excited electrons undergo a fast carrier thermalization via carrier–carrier or carrier–phonon interaction. Then thermalized carriers (hot carriers) slowly relax with a decay time beyond our measurement range (2.7 ns). The long‐lived hot carriers lifetime suggests a very weak electron–hole recombination rate in WTe_2_ due to weak phonon scattering. In addition, WTe_2_ possesses two valleys with opposite spin splitting due to time‐reversal symmetry (Figure 1b,c). Thus, it needs a momentum scattering from *k* to −*k* states for a spin depolarization. Such a process requires magnetic defects and is therefore profoundly reduced resulting in a long decay of the net spin polarization (τ_2_ ≈ 1.2 ns).

Another interesting phenomenon observed in the spin dynamics of WTe_2_ and MoTe_2_ thin film is the robust spin lifetimes against an externally applied magnetic field (*B*_ext_). The fluctuating spin–orbit coupling field *B*_So_ alone will not dephase out‐of‐plane spins of electrons since *B*_So_ orients along the *z*‐axis.^3^ The external magnetic field *B*_ext_ is applied at an angle of 60° with respect to the *z*‐axis (inset of **Figure** **4**b). Therefore, electron spins will precess about the total field, (*B*_So_ + *B_z_*_(ext)_)*z* + B*_y_*_(ext)_ *y*,^17^ where *B_z_* and *B_y_* are the components of the external magnetic field in the *zz* and *y* direction, respectively. This is similar to momentum‐dependent spin precession in conventional semiconductors such as the Dyakonov–Perel mechanism.^18^ However, Figure 4a and Figure S12 in the Supporting Information show that the Kerr signals of WTe_2_ and MoTe_2_ thin film are stable even when *B*_ext_ increases to 618 mT. The spin lifetimes extracted from biexponential fitting in both WTe_2_ and MoTe_2_ thin films slightly vary with *B*_ext_ as shown in Figure 4b. We propose that the spin stabilization in WTe_2_ and MoTe_2_ thin films originates from a large spin–orbit coupling field, which dominates over the external field. As evidenced by the theoretical calculation (Figure 1c and Figure S3c, Supporting Information), the spin–orbit splitting energy (Δ_sp_) of two lowest conduction bands reaches 30–45 meV in bilayer and 15–25 meV in 12‐monolayers WTe_2_ and MoTe_2_, which respectively corresponds to an effective field *B*_so_ of 260–389 and 130–216 T, dominating over *B*_ext_. Analytical simulations (see Note S3, Supporting Information for the details) using a modified drift–diffusion model reveal that a large spin–orbit coupling field firmly stabilizes the spin polarization along out‐of‐plane *B*_so_. As shown in Figure 4c and Figure S13 in the Supporting Information, the spin precession occurs with a small *B*_so_ (≤0.3 T). However, the spin polarization oscillation disappears with a large *B*_so_ (≥10 T) and the effect of *B*_ext_ on the spin relaxation is indeed negligible.

**Figure 4:**
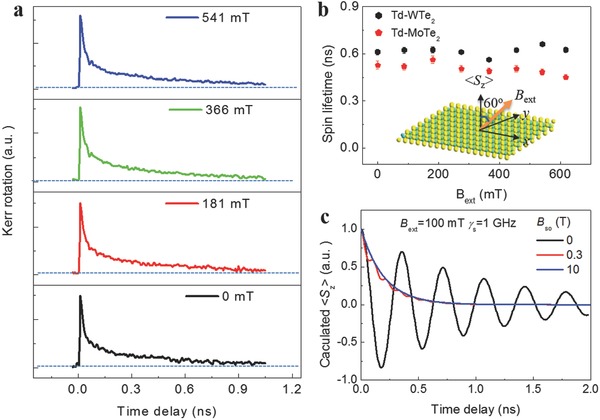
Robust spin polarization against external magnetic field. a) Kerr rotation traces of few‐layer WTe_2_ thin film with *B*_ext_. b) The spin lifetimes extracted via biexponential fitting. The Kerr rotation almost remains unchanged as *B*_ext_ increases, and the spin lifetime slightly varies with increasing *B*_ext_. The inset is the schematic diagram showing the relative orientation of *B*_ext_ and *S_z_*. *B*_ext_ is applied with an angle of 60° from the normal direction of the sample surface. c) Simulation data of spin dynamics under different spin–orbit coupling fields. γ_s_ represents the scattering rate at a given band. The obvious spin precession is observed under small *B*_so_ (≤0.3 T). However, the spin polarization ⟨*S_z_*⟩ is stabilized when *B*_so_ increases to 10 T (>>*B*_ext_ = 100 mT). The large spin–orbit coupling fields (130–389 T) in WTe_2_ and MoTe_2_ thin films firmly pin optically induced spin polarization.

We compare the spin lifetimes in WTe_2_ and MoTe_2_ thin films with topological insulator Bi_2_Se_3_. The spin lifetimes of few‐layer WTe_2_ and MoTe_2_ are three orders of magnitude longer than that of Bi_2_Se_3_ (<3 ps)^19^ at room temperature. The remarkable long‐lived spin lifetimes in WTe_2_ and MoTe_2_ thin films are the consequence of unique band structures of WTe_2_ and MoTe_2_. The WTe_2_ and MoTe_2_ show the distinct spin splitting at states (*E*, *k*) and (*E*, − *k*) with opposite spin orientations. Topological protection from time‐reversal symmetry suppresses the back momentum scattering from spin‐up (down) (*E*, *k*) states to spin‐down (up) (*E*, − *k*) states. In addition, the conduction band and valance band of WTe_2_ and MoTe_2_ are well separated in the momentum space, requiring phonons for the recombination of electrons and holes. Such a phonon‐assisted recombination lowers the recombination probability of spin‐polarized electrons and holes pairs. The low recombination probability of spin‐polarized electrons and holes is also confirmed by ultrafast reflectivity measurement, which indicates a lifetime of photoexcited electron–hole pairs longer than 2.7 ns.

In conclusion, we observe the spin lifetime of nanoseconds at room temperature in CVD‐grown few‐layer WTe_2_ and MoTe_2_ thin films using TRKR spectroscopy. We also find that the spin polarization in WTe_2_ and MoTe_2_ thin films is robust against the external magnetic field. We further discuss the underlying physics of spin dynamics in WTe_2_ and MoTe_2_ thin films. Compared with topological insulators and 2H‐TMDs, the semimetallic WTe_2_ shows a remarkably high current density,^11^ making WTe_2_ and MoTe_2_ promising for generating large charge‐to‐spin current conversion. In addition, the semimetallic nature of WTe_2_ and MoTe_2_ mitigates a current shunting issue through the ferromagnet in TMD/ferromagnet structures. Together with the long‐lived and robust spin polarization, our results suggest that WTe_2_ and MoTe_2_ may lead to novel spintronics applications.

## Experimental Section

*Synthesis and Characterization*: The few‐layer WTe_2_ and MoTe_2_ thin films were synthesized in a three‐zone CVD system (Figure S5, Supporting Information).For WTe_2_ thin film (sample 1) as a representative example, 0.3 g tungsten chloride (WCl_6_) powders (99.99%, Alfar Aesar) and 0.4 g tellurium (Te) powder (99.99%, Alfar Aesar) were placed at the first and second zone, respectively. Silicon (Si) substrates with 300 nm silicon dioxide (SiO_2_) were placed at the third zone. The furnace flowed by a mixture of 160 sccm N_2_ and 40 sccm H_2_ under ambient condition was heated to 500 °C for 20 min. Synthesis details of sample 2 is provided in Note S1 in the Supporting Information. The CVD furnace was cooled down to room temperature naturally after the growth. To prevent the samples from oxidation, all thin films were protected by a vacuum‐evaporated 1 nm Al capping which was oxidized to Al_2_O_3_ naturally in air. The samples were characterized by the HR800 Raman system (JY Horiba), AFM (SPM9700, Shimadzu Co.), XPS (AXIS‐ULTRA DLD system with an Mg KαX‐ray source), reflective OM (MV6100, Jiangnan Yongxin Co.), XRD (Empyrean, PANalytical B.V.), and TEM (Tecnai G2 F30, FEI Co.).

*Device Fabrication and Hall Transport*: The Hall devices were fabricated by two‐step photolithography. In the first step, photolithography followed by the etching using Ar ion milling was performed to define the Hall bar. In the second step, the electrodes were defined by photolithography. Finally, the Ta (4 nm)/Cu (30 nm) contacts were sputter‐deposited. The Hall transport was carried out by Quantum Design Physical Property Measurement System (PPMS) from room temperature to 2 K.

*Time‐Resolved Kerr Rotation*: The TRKR measurements were carried out using a mode‐locked Ti: Sapphire laser. The laser beam (800 nm) was split into a pump beam and a probe beam. The frequency of probe beam was doubled by second harmonic generation. The pump beam circular polarization was prepared by a succession of linear Glan–Taylor polarizer and quarter wave plate (QWP). The probe beam was linearly polarized by a Glan–Taylor polarizer. The pump beam was focused to a spot of ≈300 µm through a lens and at normal incidence with respect to the sample surface. The probe beam (spot size ≈100 µm) irradiated the sample at an angle of 10° from the normal direction of the sample surface. After reflection on the sample, the pump beam was filtered out by a long‐pass filter from the reflected probe beam. The probe beam polarization was analyzed by a succession of half wave plate (HWP), Wollaston beam splitter (WBS) and balanced photodetector (BPD). The time delay between the pump and probe beam was adjusted with a mechanical stage. To allow lock‐in measurements, the pump beam was modulated by a mechanical chopper with a frequency of 250 Hz. All the experiments were performed at room temperature. An external coil was used to apply magnetic field up to 683 mT.

*Ultrafast Reflectivity*: The transient reflectivity was carried out using a mode‐locked Ti: Sapphire laser. The femtosecond laser beam (800 nm) was split into a pump beam and a probe beam. Both the pump and probe pulses were focused onto the sample noncollinearly with spot diameters of about 300 and 100 µm, respectively. The differential reflectivity dynamics were recorded as a function of the time delay between the pump and probe pulses with crosspolarized.

*Theoretical Simulations of Spin Dynamics*: The simulations were derived from the drift–diffusion equation (Note S3, Supporting Information). The single‐valley model was used based on the band structure of WTe_2_ and MoTe_2_ and assuming a *g*‐factor of 2. The spin‐relaxation rate within the band was set to 0.5–100 GHz. *B*_so_ was divided into two distinct regions, conventional region (*B*_so_ << *B*_ext_) and stabilization region by a large spin–orbit coupling field (*B*_so_ >> *B*_ext_).

## Conflict of Interest

The authors declare no conflict of interest.

## Supporting information

Supplementary
