# Supplementary material for: Room‐Temperature Nanoseconds Spin Relaxation in WTe2 and MoTe2 Thin Films
Source: Adv Sci (Weinh). 2018 Apr 14;5(6):1700912. doi: 10.1002/advs.201700912 (PMC6010885; doi:10.1002/advs.201700912)
Supplement: Supplementary file 1 — Supplementary [file ADVS-5-1700912-s001.pdf]

Copyright WILEY-VCH Verlag GmbH & Co. KGaA, 69469 Weinheim, Germany, 2018.

ADVANCED SCIENCE
Open Access

Supporting Information

for *Adv. Sci.,* DOI: 10.1002/advs.201700912

Room-Temperature Nanoseconds Spin Relaxation in WTe_2 and $MoTe_2$ Thin Films

Qisheng Wang, Jie Li, Jean Besbas, Chuang-Han Hsu, Kaiming Cai, Li Yang, Shuai Cheng, Yang Wu, Wenfeng Zhang, Kaiyou Wang, Tay-Rong Chang, Hsin Lin, Haixin Chang, and Hyunsoo Yang**

Copyright WILEY-VCH Verlag GmbH & Co. KGaA, 69469 Weinheim, Germany, 2016.

Supporting Information

Room-temperature nanoseconds spin relaxation in WTe_2 and $MoTe_2$ thin films

Qisheng Wang, Jie Li, Jean Besbas, Chuang-Han Hsu, Kaiming Cai, Li Yang, Shuai Cheng, Yang Wu, Wenfeng Zhang, Kaiyou Wang, Tay-Rong Chang, Hsin Lin, Haixin Chang and Hyunsoo Yang**

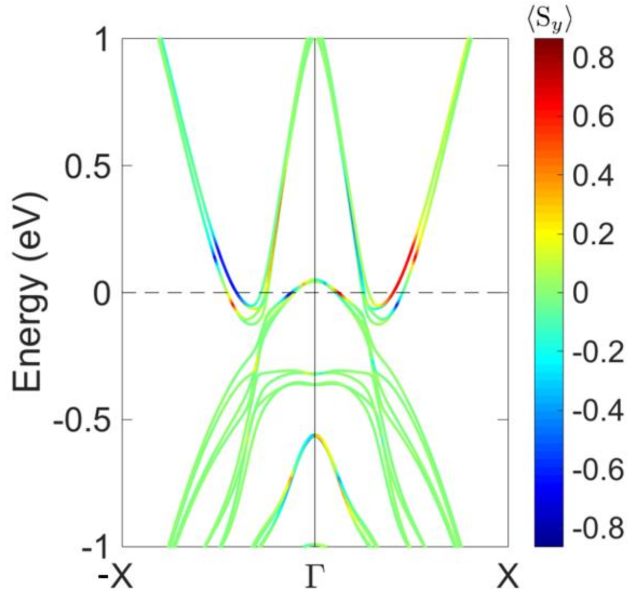

Figure S1. Spin polarization <S_y> along the *y* direction in bilayer WTe_2.

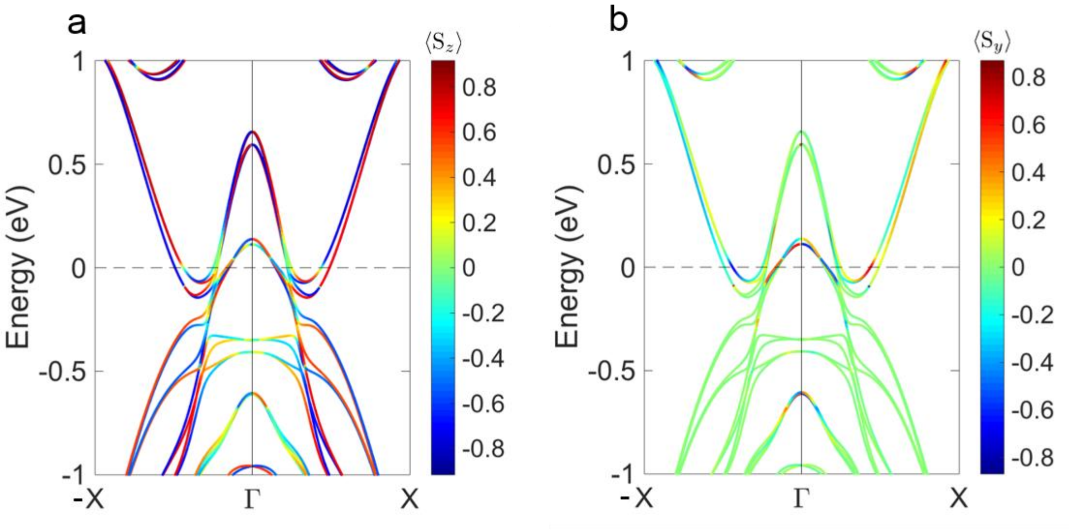

Figure S2. Spin polarization in bilayer $MoTe_2$. (a) Out-of-plane spin polarization $<S_z>$. (b) Spin polarization along the *y* direction $<S_y>$.

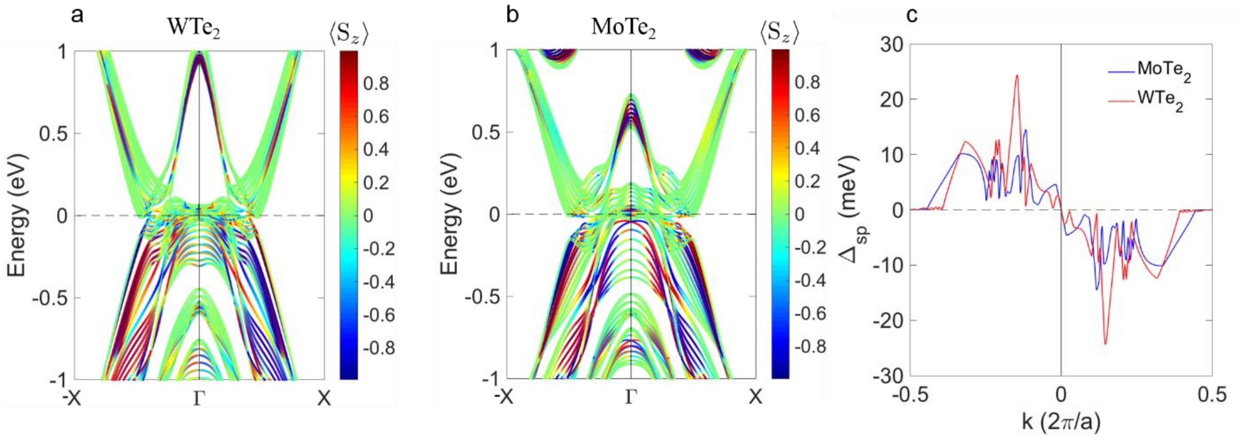

Figure S3. Out-of-plane spin polarization of twelve-monolayers (a) WTe_2 and (b) $MoTe_2$. (c) Spin splitting Δ_{sp} between spin-up and spin-down energy levels at the lowest conduction band.

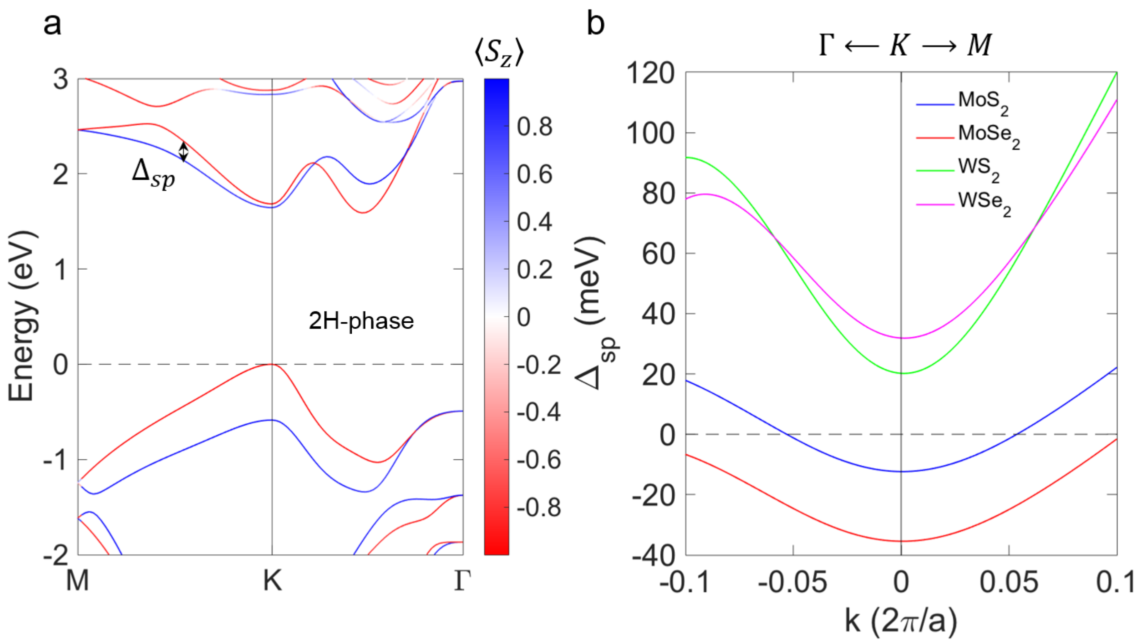

Figure S4. Spin texture and spin-orbit splitting energy in monolayer 2H-TMDs. (a) Out-of-plane spin polarization <S_z> in monolayer 2H-WSe_2. The color bar in the right label indicates the spin polarization intensity. (b) The spin-orbit splitting energy Δ_{sp} between two lowest conduction bands of monolayer MoS_2, $MoSe_2$, WS_2 and WSe_2.

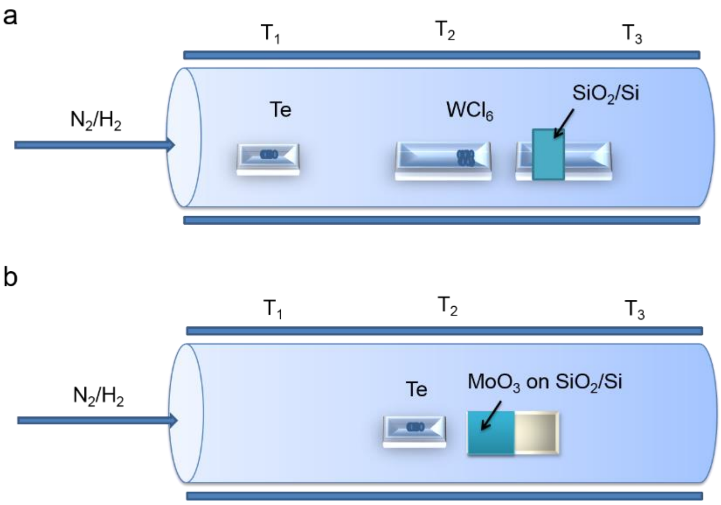

Figure S5. The CVD system for few-layer WTe_2 and $MoTe_2$ thin film preparations. Schematic diagrams of three-temperature zone furnace for synthesis of (a) few-layer WTe_2 (sample 1) using WCl_6 sources, and (b) few-layer $MoTe_2$(sample 2) applying MoO_3 thin film sources.

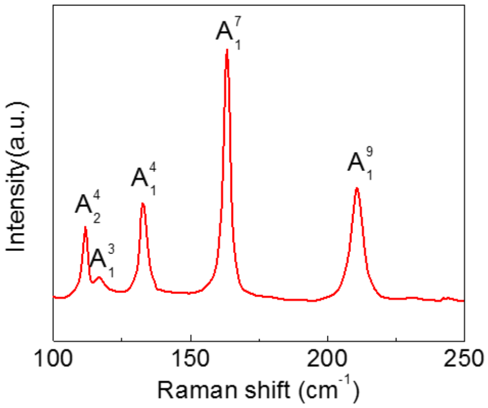

Figure S6. Raman spectra for mechanically exfoliated thin flakes from WTe_2 single crystal. The distinct peaks at 110, 117, 133, 164 and 211 cm^{-1} corresponds to A_2^4, A_1^3, A_1^4, A_1^7 and A_1^9 vibration modes, respectively.

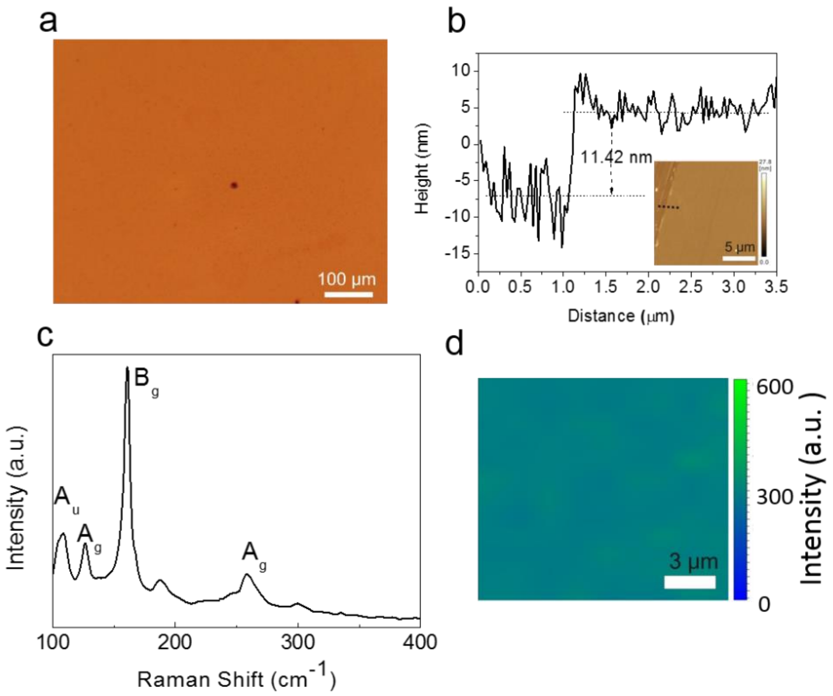

Figure S7. Characterization of few-layer $MoTe_2$ (sample 2) from MoO_3 thin film source. (a) Optical image. The thin film covers the whole substrate surface. (b) Thickness characterization of sample 2. The inset is the corresponding AFM image. The obtained film has a thickness of 11.42 nm. (c) Raman spectra displays peaks for B_g at 161.4 cm^{-1}, A_u at 106.4 cm^{-1}, A_g at 126.8 and 256.3 cm^{-1}, demonstrating the Td-phase nature of $MoTe_2$. (d)Raman mapping at B_g peak at 161.4 cm^{-1} confirms the uniformity of $MoTe_2$.

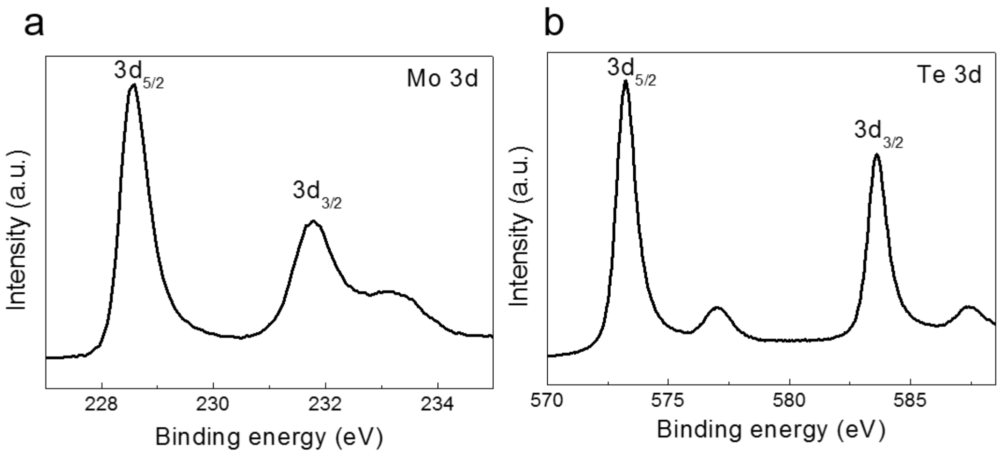

Figure S8. XPS of (a) Mo 3*d* and (b) Te 3*d* in few-layer $MoTe_2$ thin film (sample 2). The Mo $3d_{5/2}$ at 228.6 eV, $3d_{3/2}$ at 231.8 eV, Te $3d_{5/2}$ at 573.3 eV, and $3d_{3/2}$ at 583.7 eV reflect Td character of $MoTe_2$.

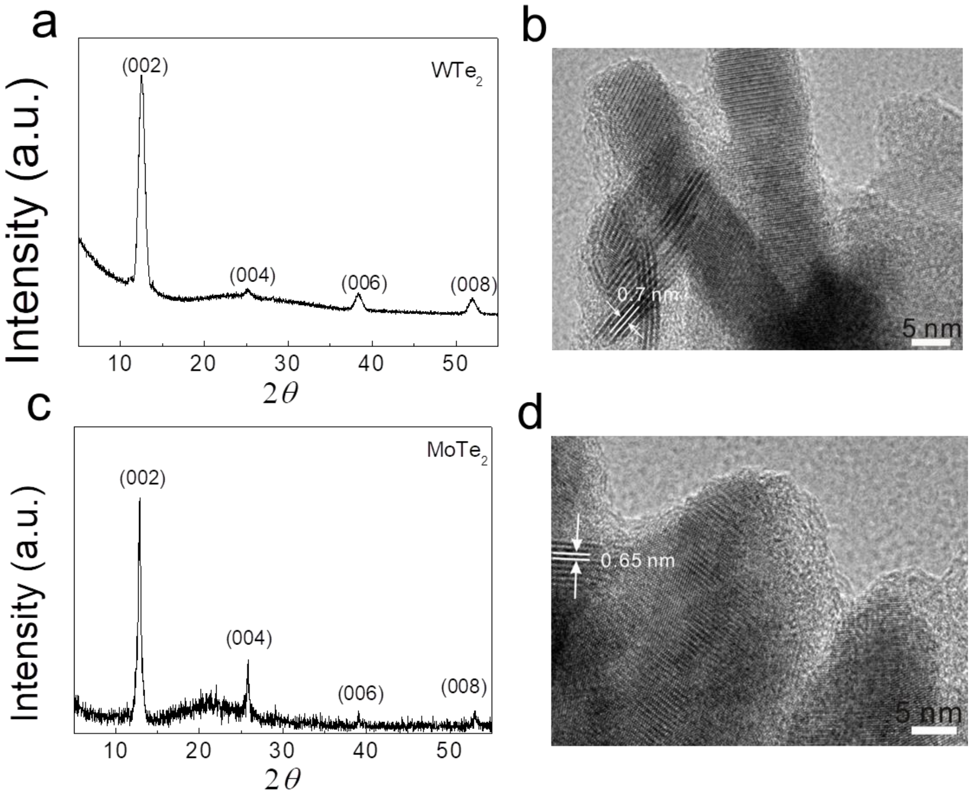

Figure S9. (a) XRD and (b) TEM of WTe_2 thin film. (c) XRD and (d) TEM of $MoTe_2$ thin film.

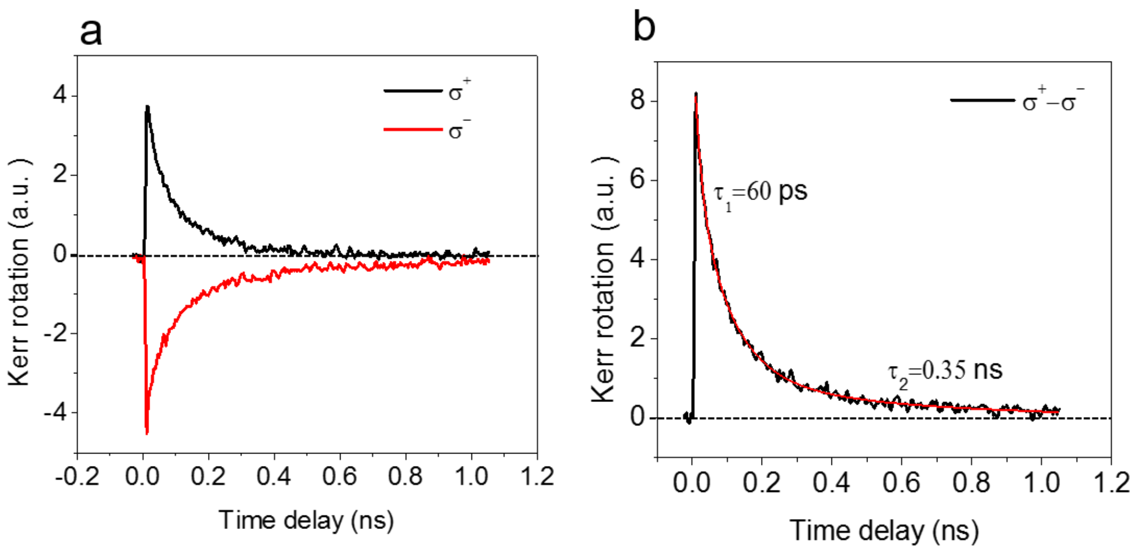

Figure S10. The TRKR data in sample 2. (a) The TRKR curve reverses the signs when the pump pulse helicity changes from σ^+ to σ^-. (b) Difference of Kerr rotation signals between σ^+ to σ^-.

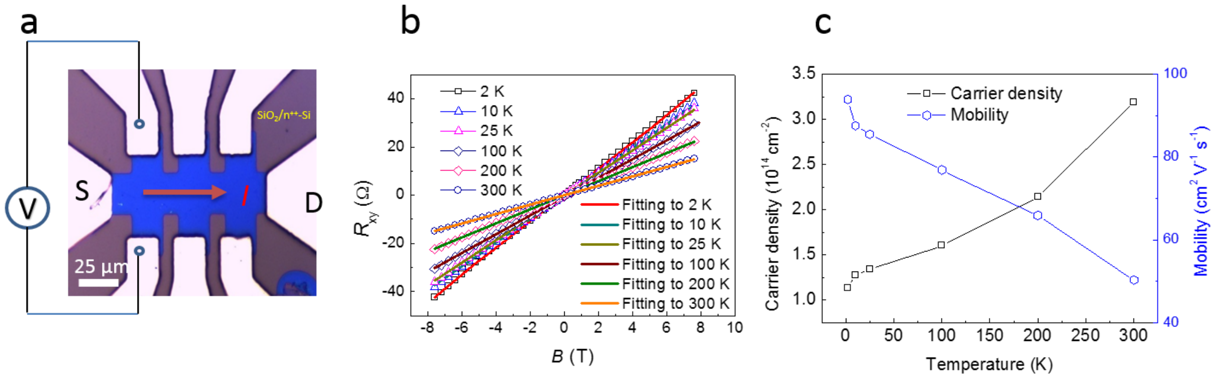

Figure S11. The Hall transport data in a typical WTe_2 thin film. (a) Optical microscopic image of a Hall device. (b) The Hall magnetoresistance (R_{xy}) versus out-of-plane magnetic field (B) from room temperature to 2 K. (c) The carrier density and mobility as the function of temperature.

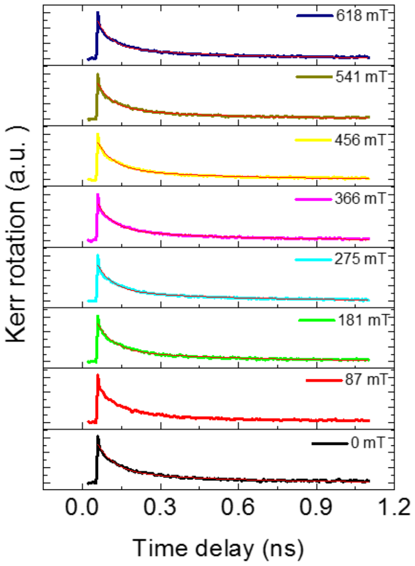

Figure S12. TRKR data as the function of pump-probe time delay under various B_{ext} in sample 2. The extracted spin lifetimes are shown in Figure 4b ($MoTe_2$).

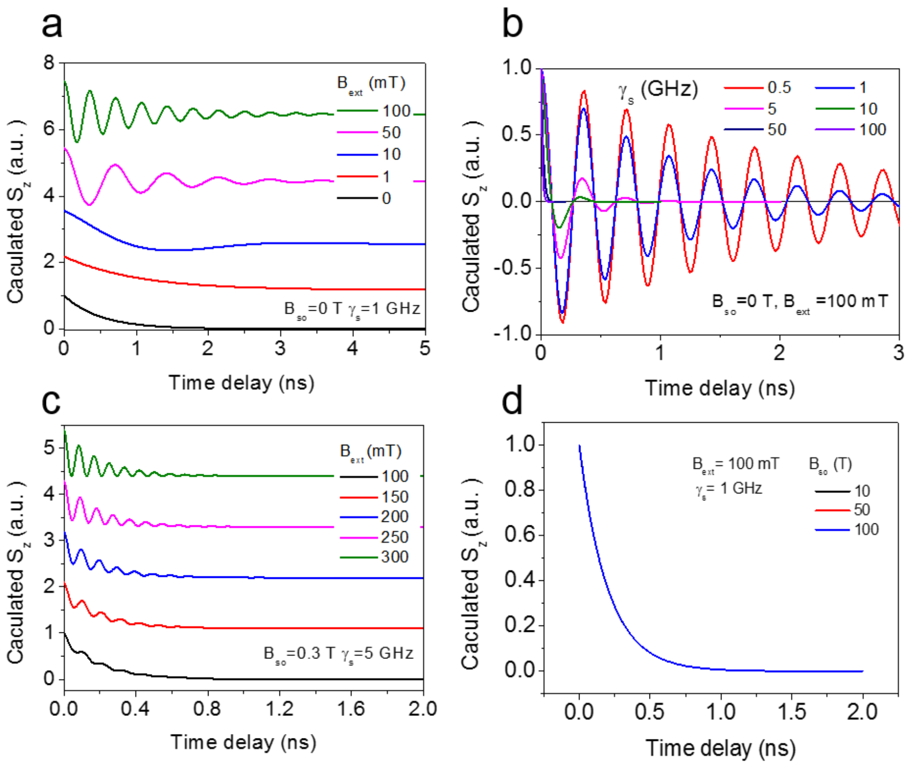

Figure S13. Theoretical simulations of spin dynamics. Two distinct regimes are considered such as $B_{so}<<B_{ext}$ and $B_{so}>>B_{ext}$. From (a), (b) and (c), we observe that when $B_{so}<<B_{ext}$, B_{ext} leads to spin precession. However, no spin precession is observed when B_{so} increases to 10 T. (d) Calculation results indicate that the robustness of the spin polarization in few-layer WTe_2 and $MoTe_2$ is due to spin stabilization from the strong spin-orbit coupling field. The model does not consider inter-valley scattering in agreement with the band structure of WTe_2 and $MoTe_2$.

Supporting Notes 1. Synthesis of sample 2

The growth was performed on a three-zone CVD system. For the growth of sample 2 (few-layer $MoTe_2$ using MoO_3 as the reaction source), a ceramic crucible loaded with 0.1 g Te powder was placed in the center of quartz tube, and the substrate with evaporated MoO_3 film was placed at the downstream about 10 cm away from Te power. A mixture of 3 sccm N_2 and 4 sccm H_2 were flowed into the tube at ambient atmosphere. The growth temperature was set to 700 °C for 30 mins. At the end of both experiments, the furnace was cooled down to room temperature naturally.

Supporting Notes 2. Analysis of Hall transport

To exclude possible artifacts in the Hall data, we have corrected the Hall resistance by subtracting the negative field trace from the positive field one. The two dimensional hole density extracted from our Hall data in Fig. S11b using one-carrier model is 3.2×10^{14} cm^{-2} at room temperature and 1.1×10^{14} cm^{-2} at 2 K. The mobility ranges from 50 cm^2 V^{-1} s^{-1} at room temperature to 94 cm^2 V^{-1} s^{-1} at 2 K. The thickness of our device obtained from atomic force microscopy (AFM) is ~4.9 nm. Due to the polycrystalline nature of our WTe_2 thin film with a typical grain size of 100 nm, the electrical transport is affected by crystal boundaries with impurities and/or defects. These disorder states are one of possible origins of holes-dominant transport.

Supporting Notes 3. Theoretical calculation of spin dynamics

We start from the drift-diffusion model to reproduce the essential feature of robust spin polarization in few-layer WTe_2 and $MoTe_2$. The spin polarization S(r) is derived from the drift-diffusion equation

$$\frac{dS(r)}{dt} = D\nabla_r^2 S(r) + \mu(E \bullet \nabla_r S(r)) + \frac{g\mu_B}{\hbar}(B \times S(r)) - \gamma_s S(r) + G(r) \quad \text{(S1)}$$

where G(r) is the generation term for optical spin injection, γ represents scattering rate, μ is the mobility, μ_B is the Bohr magneton, and g is the g-factor (g=2). The first term describes diffusion, the second term represents the drift, the third term denotes the spin precession, the fourth term is the intra-valley scattering, and the last one is the spin generation term. In this work, no electric field was applied, thus $\mu(E \bullet \nabla_r S(r))$ =0. Considering a weak pump beam induces a small spin injection, we assume the diffusion term $D\nabla_r^2 S$ is ~ 0.At initial time,

assume optically excited out-of-plane spin polarization S_z=1 (G(r) = S_z). The final description of drift-diffusion equation is given by

$$\frac{dS(z)}{dt} = \Omega_L \times S(z) + \Omega_{so} \times S(z) - \gamma_s S(z) \tag{S2}$$

where $\Omega_L = \hat{x} g\mu_B B_x / \hbar$ and $\Omega_{so} = \pm \hat{z} g\mu_B B_{so} / \hbar$ denote spin precession along B_{ext} and internal B_{so}, respectively. γ_s describes the scattering rate at a given band. If $B_{so} << B_{ext}$, the solution of Equation S2 takes the form of an exponentially decaying cosine $S(z) = S_0 \exp(-t/\tau_s)\cos(\omega_L t)$ with $\omega_L = g\mu_B B_x / \hbar$. As displayed in Figure S13a, the spin polarization sharply decays when B_{ext} goes beyond 10 mT. The obvious oscillation of spin polarization can be observed at various γ_s (Figure S13b) even if B_{so} increases to 0.3 T (Figure S13c). However, under a large B_{so}, the spin polarization is robust against B_{ext}. Figure S13d exhibits the simulated results of spin relaxation when B_{so} reaches 10, 50 and 100 T, since $B_{so} >> B_{ext}$ = 100 mT. The above calculation confirms the spin polarization is stabilized by a large B_{so} in few-layer WTe_2.

Supporting Notes 4. First principles calculations of spin texture

The first principles calculations were carried out by the VASP package[1-3] based on the projector augmented wave method within the ultrasoft pseudopotential scheme.[4,5] The generalized gradient approximation[6] was applied for the exchange-correlation interaction and the spin-orbit coupling was included. A Hamiltonian in the tight-binding formalism was built based on the Wannier functions for the bulk crystal[7] and then was applied to construct the Hamiltonians for thin film with various number of W(Mo)Te_2 layers. All these calculations used the same procedure as described in the previous work[8].

References

[1] Kresse, G., Hafner, J., Ab-Initio Molecular-Dynamics for Open-Shell Transition-Metals. Phys Rev B 48, 13115-13118 (1993).

[2] Kresse, G., Furthmuller, J., Efficient iterative schemes for ab initio total-energy calculations using a plane-wave basis set. Phys. Rev. B 54, 11169-11186 (1996).

[3] Kresse G, Furthmuller J. Efficiency of ab-initio total energy calculations for metals and semi- conductors using a plane-wave basis set. Comp. Mater. Sci. 6, 15-50 (1996).

[4] Blochl, P.E., Projector Augmented-Wave Method. Phys. Rev. B 50, 17953-17979 (1994).

[5] Kresse, G., Joubert, D., From ultrasoft pseudopotentials to the projector augmented-wave method. Phys. Rev. B 59, 1758-1775 (1999).

[6] Perdew , J.P., Burke, K., and Ernzerhof, M., Phys. Rev. Lett. 77, 3865 (1996).

[7] Mostofi, A.A., Yates, J.R., Pizzi, G., Lee, Y.S., Souza, I., Vanderbilt, D., Marzari, N., An updated version of wannier 90: A tool for obtaining maximally-localised Wannier functions Comput. Phys. Commun. 185, 2309 (2014).

[8] Chang, T.-R. et al. Prediction of an arc-tunable Weyl Fermion metallic state in $Mo_xW_{1-x}Te_2$. Nat. Commun. 7, 10639 (2016).
